# MicroRNAs in bone metastases: mechanisms and research progression

**DOI:** 10.3389/fonc.2025.1552902

**Published:** 2025-08-05

**Authors:** Wei He, Zhen Wang, ZiHan Li

**Affiliations:** Department of Orthopedics, Third Clinical School of Medicine, Ningxia Hui Autonomous Region, Ningxia Medical University, YinChuan, China

**Keywords:** bone metastases, microRNAs, breast cancer, prostate cancer, lung cancer, liver cancer

## Abstract

Bone metastasis, an exceedingly critical and often life - threatening complication in the course of diverse cancer progression, impacts nearly all tumor types, with an especially pronounced prevalence in breast, prostate, lung, liver, and thyroid malignancies. These metastatic lesions are typically localized in the spine, pelvis, shoulders, and distal femur, thereby exerting a substantial influence on patients’ quality of life and prognosis. In numerous instances, the early detection of cancers frequently coincides with patients presenting symptoms induced by bone metastases, which strikingly highlights the severe and debilitating nature of this condition. MicroRNAs (miRNAs), a class of short non - coding RNAs, have emerged as pivotal regulators of gene expression. They are widely and comprehensively acknowledged for their indispensable role in cancer initiation, progression, invasion, and metastasis. Abundant evidence has underscored the involvement of specific miRNAs at various stages of bone metastasis, further emphasizing their vital contribution to the pathogenesis of metastatic bone disease. In this comprehensive review, we systematically collate and summarize the current state - of - the - art knowledge regarding the participation of miRNAs in bone metastasis within breast, prostate, lung, liver, and thyroid cancers. Moreover, we thoroughly explore the potential of miRNAs as therapeutic targets or advanced - generation therapeutic agents for bone metastasis. Concurrently, we also in - depth investigate their function as predictive biomarkers for earlier diagnosis and the development of optimized treatment strategies for cancer patients.

## 1 Introduction

Cancer stands as the preeminent cause of mortality globally, imposing substantial economic burdens and severely compromising the quality of life (1). Bone represents a prevalent site of metastasis, afflicting a considerable number of patients with advanced - stage cancer (2). Once disseminated tumor cells (DTCs) reach the bone, they frequently enter a dormant state that can persist for intervals spanning from months to decades prior to reactivating and giving rise to detectable metastases (3). The vascular system within the bone marrow engenders fluctuations in oxygen levels, typically oscillating between 1% and 6%, rendering bone an extraordinarily hypoxic tissue (4). This hypoxic milieu sustains tumor survival under anaerobic conditions. In comparison to other tissues, the bone marrow furnishes a more propitious environment for the colonization and proliferation of metastatic cancer cells (3). This pre - metastatic bone microenvironment, also referred to as the pre - metastatic ecological niche, assumes a pivotal role in the successful implantation of cancer cells in bone (5). As previously elucidated, latent cancer cells within metastatic sites receive cues from the surrounding microenvironment, thereby triggering their proliferation (6). These cells initially form undetectable micro - metastases and subsequently evolve into clinically detectable bone metastases (7). As tumor cells metastasize to the bone microenvironment, they engage in intricate interactions with osteoblasts, osteoclasts, and bone stromal cells (8). Nevertheless, an imbalance between osteogenesis and osteoblast activity can culminate in fractures, spinal cord compression, bone pain, and disability attributable to the weakened bone structure, exerting a profound impact on both the quality of life and tumor prognosis (9). Current therapeutic modalities for bone metastases encompass systemic therapies, such as chemotherapy and endocrine therapy, designed to decelerate cancer cell proliferation; bone - targeting drugs, like bisphosphonates, which inhibit osteoclast - mediated bone resorption; novel targeted therapies, such as denosumab, that target crucial bone metabolism pathways; and bone - seeking radionuclides (10, 11). Although these treatments can enhance the quality of life for patients with bone metastases, they are predominantly palliative (12). Consequently, to forestall bone metastasis and prognosticate tumors with high metastatic potential, novel therapeutic and diagnostic strategies are urgently requisite.

MicroRNAs (miRNAs) are short non - coding RNAs that assume a pivotal role in post - transcriptional gene regulation and serve as key regulators of endogenous gene expression (13, 14). It is postulated that, in mammals, miRNAs modulate the activity of roughly 50% of all protein - coding genes. Virtually every cellular process hitherto investigated has been found to be under the regulatory influence of miRNAs, and perturbations in their expression have been implicated in a diverse array of human diseases (15). The biogenesis of miRNAs is intricate, entailing the concerted action of various enzymes to generate a mature form and to assemble the RNA - induced silencing complex (RISC) with Argonaute proteins (AGO). RISC discriminates sequences complementary to the “guide” miRNA on target mRNAs, thereby effectuating their degradation (16). Nevertheless, the post - transcriptional regulation of miRNAs within cells is extremely intricate, given that a single miRNA can target hundreds of distinct mRNAs. Moreover, the expression of a specific mRNA can be regulated by multiple miRNAs (15). The regulatory function of miRNAs is indispensable for the maintenance of normal cellular functions, and their dysregulation has been discerned in cancer cells (17). MiRNAs can function as both oncogenes and tumor suppressors in certain malignancies. Dysregulated miRNAs are known to impact cancer hallmarks such as the sustenance of proliferative signaling, circumvention of growth inhibitors, resistance to cell death, activation of invasive metastasis, and induction of angiogenesis (17, 18). Several miRNAs have been identified as instigating molecular alterations in bone metastasis. Some miRNAs are associated with aggressive cancer phenotypes that augment cell migration or with cancer cells that exhibit a greater proclivity to metastasize to bone (19). In this review, we expound upon the discovery and function of miRNAs implicated in the formation and progression of bone metastases originating from primary tumors. These findings have propelled the development of novel treatments, improved the early detection of poor prognosis, and enhanced the prediction of high - risk bone metastases.

## 2 MicroRNAs in breast cancer bone metastases

Breast cancer ranks among the most prevalent malignancies globally and has recently overtaken lung cancer as the most common cancer in women (20). In recent years, the enhanced awareness among women has spurred remarkable progress in early diagnosis and treatment. Nevertheless, metastasis persists as the primary cause of mortality in the majority of breast cancer cases (21). Bone represents the most frequent site of breast cancer metastasis (22), and the role of microRNAs (miRNAs) in breast cancer, especially in bone metastasis, has been firmly established (17).

Numerous studies have demonstrated that miRNAs regulate diverse aspects of breast cancer metastasis to bone. For example, TWIST1 (Twist Family BHLH Transcription Factor 1) promotes the expression of miR - 10b, which is usually expressed at low levels, and suppresses the expression of the transcription factor homology cassette D10, thus inhibiting bone metastasis in breastk cancer (23–26). Additionally, miR - 214 - 3p is highly expressed in breast cancer and directly targets tumor necrosis factor receptor - associated factor 3 (TRAF3), an inhibitor of osteoclast differentiation. This action facilitates osteoclast - mediated bone resorption and induces osteolytic lesions associated with bone metastases (19, 21, 27). miR - 218 - 5p is upregulated in breast cancer and acts directly on SOST and SFRP2, two inhibitors of the WNT signaling pathway, promoting bone metastasis in triple - negative breast cancer (28). miR - 940, also highly expressed in breast cancer, promotes the osteogenic differentiation of mesenchymal cells in the bone microenvironment, resulting in extensive osteoblastic differentiation and metastasis (29).

The miR - 30 family (miR - 30s), consisting of miR - 30a, miR - 30b, miR - 30c, miR - 30d, and miR - 30e, is typically downregulated in breast cancer, exerting oncogenic effects. miR - 30s inhibit bone metastasis by negatively regulating key oncogenes and osteoclast growth factors (e.g., IL - 8, IL - 11, DKK - 1, RUNK2, CDH11, CTGF, and integrins ITGA5 and ITGB3) that influence bone metastasis (30). miR - 34a, which is downregulated in triple - negative breast cancer (TNBC) — the most lethal subtype of breast cancer (31, 32) — inhibits tumor growth. Upregulation of miR - 34a promotes proliferation and invasion, activates cellular senescence, and increases sensitivity to BCL - ABL and SRC tyrosine kinase inhibitors, such as dasatinib (33).

Furthermore, downregulation of miR - 124 leads to elevated levels of its direct target, IL - 11, which acts as an effector to promote osteogenesis and bone metastasis formation. This modifies the bone microenvironment, rendering tumors more prone to metastasize to bone (8). The forced expression of miR - 125b has been shown to impede the development of bone metastasis by downregulating Ets1, a key factor in the invasive program of cancer cells. This downregulation also diminishes the extent of osteolytic damage *in vivo*. miR - 125b, when combined with the chemotherapeutic agent NS - 398, augments its inhibitory effect in animal models, possibly by influencing the interaction of ETS1 with HIF - 1α, thereby impairing PTGS2 (34). High expression of miR - 135 and miR - 203 in highly bone - invasive breast cancer cell lines, such as MDA - MB - 231, inhibits migration *in vitro* by downregulating genes associated with cell motility (ROCK, CD44, and PTK2) (35). miR - 135 also targets RUNX2 in MDA - MB - 231 cells, safeguarding against osteolytic lesion formation in metastatic animals (35). Additionally, miR - 135 targets Smad5, a component of the BMP signaling pathway, thereby reducing the expression of ID2, a gene downstream of BMP - 2, to inhibit bone metastasis (35).

Furthermore, miR - 429 is downregulated in the bone tissue of metastatic breast cancer patients compared to primary tumor tissue, and its low expression is positively correlated with poor bone metastasis - free survival in breast cancer patients. miR - 429 promotes osteoprotegerin (OPG) expression in MC3T3 - E1 osteoblasts and reduces the expression of RANKL (osteoblast differentiation factor), indicating its role in promoting osteogenic differentiation *in vitro* (36). Moreover, miR - 429 inhibits osteoblast differentiation by directly targeting CRKL (V - CRK avian sarcoma virus CT10 - homolog - like) and reducing MMP - 9 expression, thereby inhibiting bone metastasis (36).

MicroRNA - based therapies have shown promise in the context of breast cancer genesis and metastasis. However, challenges persist due to potential adverse effects. Continued research into new miRNA - targeted agents and their downstream effects holds significant therapeutic potential for the future (8) (**Table 1**).

**Table 1:** Expression of microRNAs in breast cancer.

MicroRNAs	Expression	Promotes/Inhibits	Targets/Pathways
miR-10b	High	–	TWIST1
miR-214-3p	High	+	TRAF3
miR-940	High	+	
miR-218-5p	High	+	WNT signaling pathway
MiR-30s	Low	–	IL-8, IL-11, DKK-1, RUNK2, CDH11, CTGF, ITGA5, ITGB3
miR-34a	Low	–	BCL-ABL
miR-124	Low	+	IL-11
miR-125b	Low	–	ETS1, HIF-1α
miR-135		–	RUNX2, Smad5
miR-203	High	–	ROCK, CD44, PTK2
miR-429	Low	+	OPG/RANKL
miR-429	Low	+	CRKL

## 3 MicroRNAs in prostate cancer bone metastases

Prostate cancer (PCa) ranks as the most prevalent cancer in men and stands as the leading cause of cancer - related mortalities (37). The prognosis and clinical outcomes of prostate cancer are directly contingent upon the occurrence of metastases. The bone microenvironment serves as a propitious ecological niche for metastasis (38), with approximately 70% of prostate cancer patients ultimately developing bone metastases (39). MicroRNAs (miRNAs) have long been recognized as potential biomarkers for prostate cancer (40), presenting the possibility of actionable guidance for risk assessment and the advancement of personalized medicine (41).

Accumulating evidence shows that miR - 152 - 3p is downregulated in prostate cancer. Prostate cancer - derived small extracellular vesicles (SEVs) carrying miR - 152 - 3p transmit osteolytic signals from tumor cells to osteoblasts, promoting the osteolytic progression of bone metastases and modulating osteoclast activity by acting on the regulatory factor MAFB produced by osteoclasts (42). Meanwhile, miR - 210 - 3p is elevated in bone metastatic prostate cancer tissues compared to non - bone metastatic ones. Its upregulation enhances epithelial - mesenchymal transition (EMT), invasion, and migration of prostate cancer cells by sustaining the activation of NF - κB signaling, achieved by targeting TNIP1 and SOCS1 (43).

MicroRNAs like those from the miR - 200 family, miR - 205, and miR - 203, generally underexpressed in prostate cancer, promote epithelial transformation and inhibit metastasis by suppressing EMT through targeting EMT transcription factors (EMT - TFs) (44). In contrast, the DLK1 - DIO3 family of miRNAs, including miR - 409 - 3p/5p, miR - 154*, and miR - 379, are upregulated in prostate cancer and play a role in promoting bone metastasis (45).

miR - 145, lowly expressed in prostate cancer, can suppress both tumor growth and bone metastasis by inhibiting MYC and RAS, and has been shown to retard metastasis in animal models (46). Similarly, high expression of miR - 146a in prostate cancer patients with bone metastases implies its potential to promote metastasis and invasion (47). Moreover, the loss of miR - 15 and miR - 16, along with an increase in miR - 21, synergistically promotes the spread of prostate cancer and bone damage, endowing prostate cancer cells with the potential for bone metastasis by aberrantly activating TGF - β and Hedgehog signaling (48).

miR - 378a - 3p, downregulated in prostate cancer, induces osteolytic progression through the activation of the DYRK1A/NFATC1/AngPTL2 axis in bone marrow macrophages (BMMs), playing a crucial role in promoting bone metastasis and potentially serving as a predictor of metastatic prostate cancer (49). Transcriptome analysis of prostate cancer - derived extracellular vesicles (EVs) has identified the enrichment of miR - 26a - 5p, miR - 27a - 3p, and miR - 30e - 5p, which are involved in BMP - 2 - induced osteogenic inhibition *in vivo* and seem to play a pivotal role in prostate cancer - mediated inhibition of osteoblast activity (50).

miR - 181b - 5p, downregulated in prostate cancer, directly targets the OSM 3’UTR, inhibits OSM mRNA and protein expression, decreases IL - 6 and AREG, and increases OPG, leading to the inhibition of bone metastatic activity in prostate cancer (51). miR - 135b, significantly downregulated in prostate cancer, promotes bone metastasis by enhancing the migration capacity of prostate cancer cells, with PLAG1, JAKMIP2, PDGFA, and VTI1B identified as potential mediators (52).

Low expression of miR - 127 - 3p promotes prostate cancer cell invasion and migration *in vitro* by targeting the proteasome β subunit PSMB5. CTCF transcriptionally represses miR - 127 - 3p by interacting with its promoter, activating the CTCF/miR - 127 - 3p/PSMB5 axis to promote prostate cancer bone metastasis (53). miR - 141 - 3p is downregulated in bone metastatic prostate cancer tissues, and its low expression is positively correlated with serum PSA levels, Gleason grade, and bone metastasis status. Upregulation of miR - 141 - 3p markedly reduces bone metastasis *in vivo* by inhibiting NF - κB signaling through directly targeting TRAF5 and TRAF6 (54).

Reduced expression of miR - 133a - 3p in prostate cancer tissues, especially in bone metastatic tissues, is significantly associated with advanced clinicopathological features and shorter metastasis - free survival. miR - 133a - 3p inhibits PI3K/AKT signaling by directly targeting multiple cytokine receptors (e.g., EGFR, FGFR1, IGF1R, and MET), thereby inhibiting bone metastasis (55). miR - 466, also downregulated in prostate cancer tissues, inhibits tumor growth and bone metastasis by directly regulating the osteogenic transcription factor RUNX2 (56).

Notably, a substantial number of prostate cancer - related microRNAs have been reported, many of which directly impact the prognosis of prostate cancer and bone metastasis. This discovery offers an exciting future direction for the identification of new therapeutic targets for prostate cancer bone metastasis (**Table 2**).

**Table 2:** Expression of microRNAs in prostate cancer.

MicroRNAs	Expression	Promotes/Inhibits	Targets/Pathways
miR-152-3p	Low	+	MAFB
miR-210-3p	High	+	EMT, NF-κB signaling pathway
miR-200s		–	EMT
DLK1-DIO3 family	High	+	
miR-146a	High	+	MYC, RAS
miR-15, miR-16		+	TGF-β
miR-378a-3p	Low	+	DYRK1A/NFATC1/AngPTL2 axis
miR-26a-5p, miR-27a-3p, miR-30e-5p			
miR-181b-5p	Low	–	3’UTR
miR-135b	Low	+	PLAG1, JAKMIP2, PDGFA, VTI1B
miR-127-3p	Low	+	PSMB5
miR-127-3p	Low	+	CTCF/miR-127-3p/PSMB5 axis
miR-141-3p	Low	+	NF-κB signaling pathway
miR-133a-3p	Low	–	PI3K/AKT signaling pathway
miRNA-466	Low	–	RUNX2

## 4 MicroRNAs in lung cancer bone metastases

Lung cancer ranks among the most frequently diagnosed cancers worldwide and is the preeminent cause of cancer - related mortalities. Annually, it is estimated that there are approximately 2 million new cases and 1.76 million deaths (57). Despite remarkable advancements in treatment modalities, the incidence of lung cancer persists in its upward trend, and the mortality rate remains alarmingly high (58). Treatment strategies diverge according to the stage of cancer, yet these interventions have exerted a rather limited impact on reducing mortality (59, 60). Bone metastases, which are frequently associated with severe skeletal - related events, represent the most common site of distant tumor dissemination and can be detected in over one - third of patients with advanced lung cancer (61). MicroRNAs have been recognized as pivotal biomarkers in bone metastasis originating from lung cancer (61).

Exosomes derived from hypoxic bone marrow - derived mesenchymal stem cells (BMSCs) transfer miR - 193a - 3p, miR - 210 - 3p, and miR - 5100 to adjacent cancer cells. This transfer promotes invasion and bone metastasis through the activation of STAT3 signaling and epithelial - mesenchymal transition (EMT) (62). Additionally, non - small cell lung cancer (NSCLC) - derived lncRNA - SOX2OT regulates osteoblast differentiation and stimulates bone metastasis by targeting the miRNA - 194 - 5p/RAC1 axis and the TGF - β/PTHRP/RANKL signaling pathway in osteoblasts (63).

The elevated expression of miR - 106a in lung cancer bone metastases propels metastatic progression by targeting tumor protein 53 - induced nuclear protein 1 (TP53INP1), thereby enhancing cell migration, autophagy - dependent cell death, and EMT (64). In lung adenocarcinoma, the expression of miR - 21 is upregulated. Exosomal miR - 21 promotes osteoclastogenesis by targeting programmed cell death factor 4 (PDCD4), a well - known regulator of osteoclast differentiation, and consequently promotes bone metastasis (65, 66).

Exosomal miR - 17 - 5p is also upregulated in non - small cell lung cancer cell lines with bone metastasis when compared to primary cell lines. MiR - 17 - 5p targets PTEN, promoting osteoclastogenesis and bone metastasis via the PI3K/AKT signaling pathway (67). Moreover, miR - 192 - 5p is downregulated in lung cancer tissues, and its decreased expression is correlated with increased TRIM44 levels, which are associated with augmented proliferation, migration, and invasion of lung cancer cells. Studies have demonstrated that miR - 192 - 5p inhibits lung cancer progression and bone metastasis by negatively regulating TRIM44 (68).

In summary, an increasing volume of evidence accentuates the crucial role of microRNAs in lung cancer bone metastasis. Some of these microRNAs have been identified as indicators of bone metastasis risk. However, a vast expanse remains to be explored concerning their therapeutic potential (**Table 3**).

**Table 3:** Expression of microRNAs in lung cancer.

MicroRNAs	Expression	Promotes/Inhibits	Targets/Pathways
miR-193a-3p, miR-210-3p, miR-5100		+	STAT3
miRNA-194-5p		+	EMT, NF-κB signaling pathway
miR-106a	High	+	EMT
miR-21	High	+	
miR-17-5p	High	+	MYC, RAS
miR-192-5p	Low	–	TGF-β

## 5 MicroRNAs in liver cancer bone metastases

Liver cancer ranks as the fifth most prevalent cancer and the fourth leading cause of cancer - related mortality globally (69). Hepatocellular carcinoma (HCC) represents the most common type of liver cancer and is a primary cause of cancer - related deaths in numerous regions (70). Owing to the high clinical and biological heterogeneity of HCC tumors, liver cancer is frequently diagnosed at an advanced stage. Currently, microRNAs are being extensively explored as tools for early detection, prognostic prediction, and novel therapeutic targets in liver cancer (71).

miR - 34a, which is downregulated in HCC compared to normal liver tissue, has been demonstrated to influence bone metastasis by targeting Smad4 via the TGF - β pathway. This action affects the transcription of downstream genes associated with bone metastasis, such as CTGF and IL - 11 (72). Reduced expression levels of miR - 34a in serum and HCC tissues have been identified as independent risk factors for the development of bone metastasis. Moreover, miR - 34a expression levels can serve as a predictor of bone metastasis in HCC patients (73).

miR - 424 - 5p expression is decreased in HCC tissues and cell lines and is correlated with AFP levels, TNM stage, intrahepatic metastasis, and poor overall survival. miR - 424 - 5p directly targets TRIM29, a gene that inhibits cell proliferation and invasion, and is associated with HCC cell proliferation and invasion (74). Similarly, the expression of miR - 708 is significantly reduced in HCC tissues compared to adjacent non - cancerous tissues. Low miR - 708 expression is closely associated with higher Edmondson - Steiner grades and advanced tumor lymph node metastasis. Additionally, the forced expression of miR - 708 inhibits the migration and invasion of HCC cell lines *in vitro* (75).

miR - 16 exhibits the highest expression in SMMC - 7721 cells and the lowest in SK - Hep - 1 and Huh - 7 cells. Overexpression of miR - 16 in HepG2 cells inhibits proliferation, invasion, and metastasis, and these effects are associated with the PI3K/Akt signaling pathway (76). Moreover, miR - 26a is significantly downregulated in HCC tissues, where it directly targets the 3’UTR of FBBXO11 mRNA, inhibiting its expression and promoting the proliferation, colony formation, migration, and invasion of HCC cells (77).

miR - 1296 is also reduced in HCC tissues and cell lines. It negatively regulates SRPK1 by binding to its 3’UTR, leading to the inhibition of P - AKT. Re - expression of SRPK1 or activation of the PI3K/AKT pathway partially reverses the effects of miR - 1296 on HCC cell migration, invasion, and epithelial - mesenchymal transition (EMT). miR - 1296 inhibits HCC metastasis and EMT progression by targeting the SRPK1 - mediated PI3K/AKT pathway (78).

Notably, research has revealed that microRNAs play a significant role in the invasive metastasis of hepatocellular carcinoma. Their regulatory mechanisms overlap with pathways that regulate bone metastasis in breast and prostate cancers, such as NF - κB, PI3K - AKT, and EMT. These findings imply that there is substantial potential for further exploration of microRNAs in HCC bone metastasis, which could open up new avenues for high - risk screening and early diagnosis (**Table 4**).

**Table 4:** Expression of microRNAs in liver cancer.

MicroRNAs	Expression	Promotes/Inhibits	Targets/Pathways
miR-34a	Low	–	TGF-β
miR-424-5p	Low	+	TRIM29
miR-708	Low	–	
miR-16	High/Low	–	PI3K/Akt signalling pathway
miR-26a	Low	+	3’UTR of FBXO11 mRNA
miR-1296	Low	–	SRPK1, PI3K/Akt pathway

## 6 MicroRNAs in other osteometastases

Thyroid cancer constitutes approximately 3% of all cancers globally, with around 5.86 million new cases documented in 2020 (79). Over the past three decades, the incidence of thyroid cancer has witnessed a remarkable increase in several high - and middle - income countries (80). Nevertheless, in recent years, the mortality rate of thyroid cancer has either decreased or remained stable, and it is generally low in most regions (81). This can be largely ascribed to the advancements in diagnostic techniques and treatment modalities (82).

In the case of a male patient diagnosed with follicular thyroid cancer and distant metastases to the eleventh thoracic vertebra, who underwent total thyroidectomy and resection of metastatic lesions, postoperative localized spinal metastases were found to be significantly associated with the low expression of miRNA - 486 - 5p. This low expression was correlated with a poor prognosis, and miR - 486 - 5p was discovered to have an impact on epithelial - mesenchymal transition (EMT) (83–85).

Regarding osteosarcoma, an upregulation of miR - 25 - 3p has been observed. MiR - 25 - 3p is secreted by osteosarcoma cell lines and detectable in tumor - derived exosomes, with high serum concentrations being correlated with a poor prognosis in osteosarcoma patients (86). Moreover, miR - 30a is significantly underexpressed in osteosarcoma cell lines, and it can target RUNX2, thereby inhibiting the proliferation, migration, and invasion of osteosarcoma cells (87). Additionally, miR - 603 expression is markedly upregulated in human osteosarcoma tissues and cell lines, where it exerts oncogenic effects by inhibiting BRCC2. Meanwhile, miR - 143 - 3p inhibits osteosarcoma cell proliferation and metastasis while promoting apoptosis by targeting FOS - like antigen 2 (FOSL2) (88, 89).

In Ewing sarcoma, both miR - 124 - 3p and miR - 139 - 5p are significantly downregulated. The reduced expression of miR - 124 - 3p is associated with poor survival, while the overexpression of miR - 139 - 5p has shown inconsistent effects: upregulation of miR - 139 - 5p significantly reduced cell invasion but increased the clonogenic capacity *in vitro* (90).

Despite the fact that numerous tumors have the potential to develop bone metastases, the expression of microRNAs in bone metastases from primary tumors, apart from those previously mentioned, remains relatively under - investigated (**Table 5**).

**Table 5:** Expression of microRNAs in other cancers.

MicroRNA	Cancer type	Expression	Promote/Inhibit	Targets/Pathways
miR-486-5p	Follicular Thyroid Carcinoma	–	+	EMT
miR-25-3p	Osteosarcoma	High	+	–
miR-30a	Osteosarcoma	Low	–	RUNX2
miR-603	Osteosarcoma	High	+	BRCC2
miR-143-3p	Osteosarcoma	Low	–	FOSL2
miR-124-3p, miR-139-5p	Ewing’s Sarcoma	Low	+	–

## 7 Future

A plethora of studies in the literature unequivocally demonstrate that bone metastases are a phenomenon occurring in nearly all cancer types. Nevertheless, it has been discerned that breast, prostate, lung, liver, and thyroid cancers exhibit a higher proclivity for developing bone metastases compared to other malignancies. Despite the substantial body of research, a significant number of microRNAs in various cancers remain uncharted territory, thereby necessitating further in - depth investigation. Bone metastasis is intricately associated with alterations in the skeletal microenvironment, particularly the pre - metastatic niche, where diverse regulators contribute to the disruption of the balance between osteolysis and osteogenesis. A multitude of molecules have been observed to undergo changes within the metastatic microenvironment.

Notably, although microRNAs have been extensively explored as novel detection biomarkers, prognostic factors, and potential therapeutic targets in hepatocellular carcinoma (HCC) and thyroid cancers, the specific role of microRNAs in bone metastasis within these cancers remains inadequately explored. Encouragingly, microRNAs associated with cancer progression, invasion, and metastasis have been identified in both hepatocellular carcinoma and thyroid cancer. Intriguingly, the regulatory pathways of these microRNAs involved in cancer progression and metastasis overlap with those previously implicated in bone metastasis. Based on this convergence, it is postulated that microRNAs enriched in these cross - regulatory pathways in HCC and thyroid cancer might be involved in bone metastasis, thus presenting an exciting area for further exploration.

MicroRNAs have been incontrovertibly shown to play pivotal roles in the development of diverse tumors, exerting influence on different microenvironments and molecular pathways. Among the extant reports, it is evident that microRNAs derived from extracellular vesicles or tumor - derived microRNAs assume diverse functions, either acting as pro - oncogenic factors that promote tumor invasion and metastasis or as tumor suppressors that inhibit tumor progression. Additionally, the expression levels of specific microRNAs exhibit variability across different tumors, underscoring the imperative need for further research into their precise molecular mechanisms.

**Figure 1:**
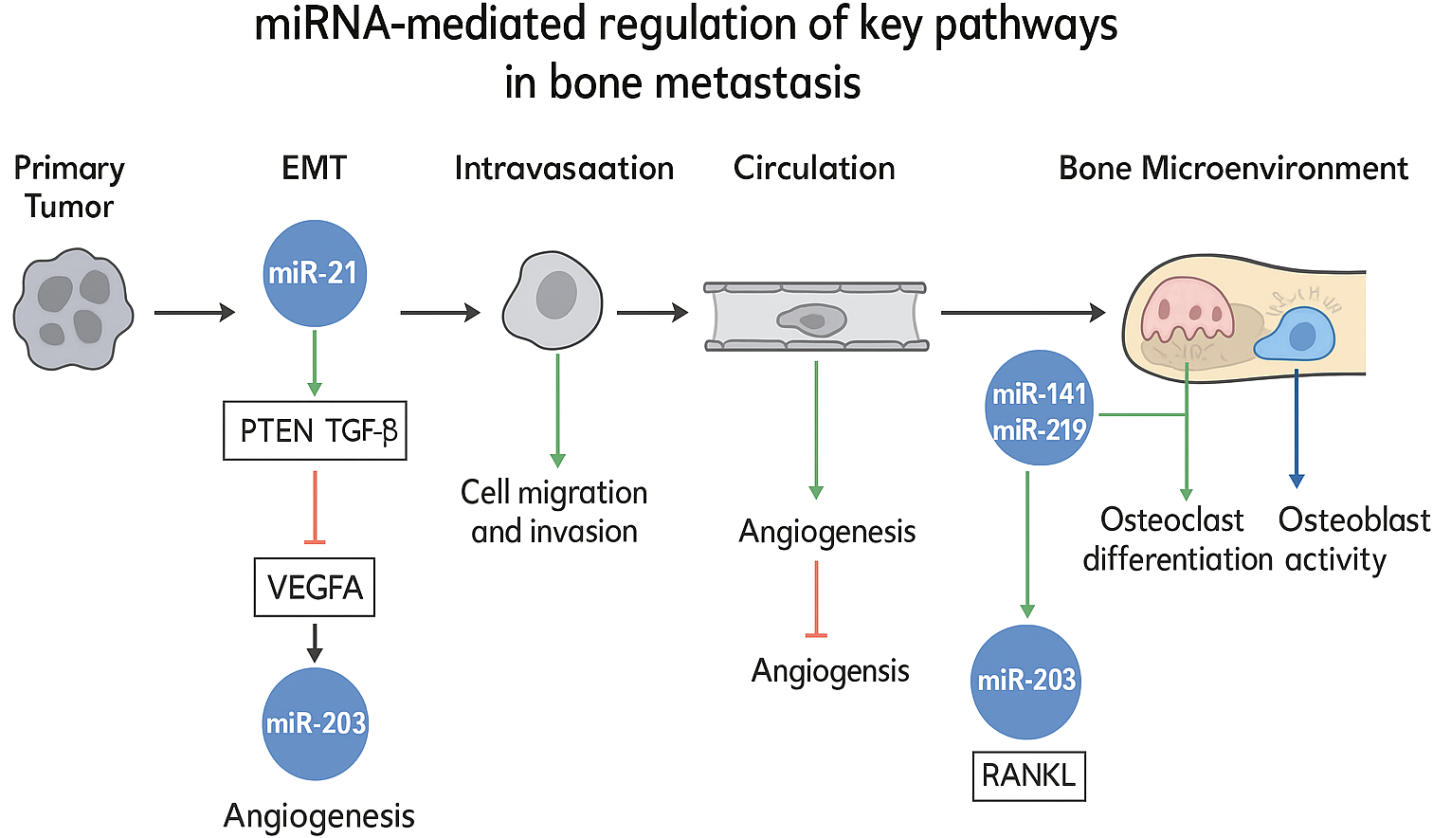
miRNA-mediated regulation of key pathways in bone metastasis.

In the context of bone metastasis, microRNAs have been found to be highly enriched in key signaling pathways, such as NF - κB, PI3K - AKT, and Wnt. This not only corroborates the critical role of microRNAs in tumor development and bone metastasis but also accentuates their potential as diagnostic tools. To integrate these multifaceted interactions, Figure X schematically delineates the mechanistic roles of specific miRNAs in regulating essential pathways—including epithelial-mesenchymal transition (EMT), angiogenesis, and osteoclast/osteoblast dynamics—within the bone metastatic cascade. This visual synthesis highlights miRNA-mediated crosstalk between disseminated tumor cells and the bone microenvironment, elucidating their functional contributions to metastatic progression. (**Figure 1**) Currently, several highly specific microRNAs are being actively investigated as target molecules for clinical risk assessment in breast and prostate cancer. This not only suggests promising directions for the discovery of new therapeutic targets but also validates the therapeutic potential of microRNAs.

Despite promising preclinical outcomes, the clinical translation of miRNA-based therapeutics faces substantial barriers. Key obstacles include: (i) Physiological delivery barriers, such as enzymatic degradation, renal clearance, and inadequate tumor penetration, which limit bioavailability; (ii) Off-target effects stemming from partial complementarity to non-cognate mRNAs, potentially inducing unintended transcriptome-wide perturbations; (iii) Immune activation triggered by carrier materials or miRNA mimics, provoking systemic inflammatory responses; and (iv) Instability in circulation due to nuclease susceptibility, necessitating complex chemical modifications. These collective challenges underscore the imperative for advanced delivery platforms capable of spatial-temporal control.

Indeed, the utilization of encapsulated microRNA analogs as cancer treatments has been extensively studied, with notable therapeutic responses observed in certain cases. However, challenges persist, including adverse effects that have led to the termination of specific treatments. Urgent development of new delivery methods and strategies is required to surmount these obstacles and enhance the efficacy of microRNA - based therapies. Nevertheless, translational progress remains limited: despite over 30 miRNA-targeting agents entering Phase I/II oncology trials since 2016, none have achieved regulatory approval specifically for bone metastases. This disparity arises from context-dependent miRNA functionality—where a single miRNA may exhibit pro-metastatic or tumor-suppressive roles across different microenvironments—compounded by inadequate biomarker-guided patient stratification. Additionally, the unique vascular and hypoxic nature of bone niches impedes uniform drug distribution, while the absence of validated surrogate endpoints for bone metastasis further complicates clinical trial design. Bridging this translational chasm demands coordinated efforts in three domains (1): developing miRNA signatures predictive of bone tropism (2); engineering niche-specific delivery systems; and (3) establishing standardized response metrics for osseous lesions.”

In summary, the role of microRNAs in bone metastasis is indisputable, and substantial progress has been achieved in understanding their involvement. Nevertheless, the diagnostic and therapeutic implications of microRNAs in bone metastasis still demand further exploration. MicroRNAs are expressed in a wide array of tumors with diverse functions, even within the same tumor type. Looking ahead, future efforts will be centered on identifying highly specific diagnostic and prognostic microRNA targets, as well as uncovering new therapeutic approaches. These advancements will be instrumental in improving the diagnosis and treatment of bone metastasis.
